# Study on Delamination Between Polymer Materials and Metals in IC Packaging Process

**DOI:** 10.3390/polym11060940

**Published:** 2019-05-30

**Authors:** Cheng-Tang Pan, Shao-Yu Wang, Chung-Kun Yen, Chien-Kai Ho, Jhan-Foug Yen, Shi-Wei Chen, Fan-Rui Fu, Yi-Tzu Lin, Cing-Hao Lin, Ajay Kumar, Yow-Ling Shiue

**Affiliations:** 1 Department of Mechanical and Electro-Mechanical Engineering, National Sun Yat-sen University, Kaohsiung 804, Taiwan; pan@mem.nsysu.edu.tw (C.-T.P.); sywang@mem.nsysu.edu.tw (S.-Y.W.); alden0113@gmail.com (C.-K.Y.); chienkai6487@gmail.com (C.-K.H.); ajaynsysu@mem.nsysu.edu.tw (A.K.); 2 Institute of Medical Science and Technology, National Sun Yat-sen University, Kaohsiung 804, Taiwan; 3 Advanced Semiconductor Engineering Inc., Kaohsiung 811, Taiwan; Mike_Yen@aseglobal.com (J.-F.Y.); DarrenSW_Chen@aseglobal.com (S.-W.C.); Mars_Fu@aseglobal.com (F.-R.F.); Iisa_Lin@aseglobal.com (Y.-T.L.); Jam_Lin@aseglobal.com (C.-H.L.); 4 Institute of Biomedical Sciences, National Sun Yat-sen University, Kaohsiung 804, Taiwan

**Keywords:** LFBGA, PBGA, delamination, contact angle, XPS, FEA

## Abstract

The electronic package interconnects electronic signals from one area to another and package delamination is a serious problem in the configuration of materials. This study focused on decreasing the delamination of the low-profile fine pitch ball grid array (LFBGA) and plastic ball grid array (PBGA) packages in terms of polymer thermal issue, metal bonding and bonding mechanisms. PBGA and LFBGA are a very common type of packaging processes in the electronics industry. The present study dealt first with delamination of the LFBGA packaging, through characterization and determination of physical and chemical properties such as surface roughness, surface energy, and contact angle. The relationship between surface roughness and delamination was verified through various roughness bonding experiments. In addition, the surface energy was determined by measuring the contact angle after cleaning the metal surface of Cu, Ni and Cr with Ar + O_2_ gas, and, this gas plasma treatment was applied to enhance the adhesive properties. The compositions of the surface were analyzed through an X-ray photoelectron spectroscopy (XPS). Also, the delamination issue between the corner of the heat sink cap and the epoxy resin was observed for delamination of the LFBGA packaging. Further, this study analyzed the PBGA packaging process through the finite element analysis simulation software ANSYS. To improve the heat sink cap delamination issue of the PBGA, a new chamfer design of the corner seat was streamlined to decrease the stress value and delamination. Besides, the simulation results demonstrated that the stress value reduced after increasing the shoulder length. The results implicate that the stress value is inversely proportional to the shoulder width and the chamfer radius. This study demonstrated that the optimization in design was able reduce the delamination phenomena in configuration material.

## 1. Introduction

Since Texas Instruments (TI) developed integrated circuits (IC), numerous and vigorous studies in the field of semiconductors have been performed. The global market share of the Taiwanese semiconductor industry is increasing annually due to government support and active academic studies. Taiwan plays an active and important role in the IC design and packaging industry. This has been a focus of research and development in the semiconductor industry over the past two decades. The entire process requirement for the industry is available, from wafer materials to IC design, manufacturing, packaging and system testing. Ball grid array (BGA) packaging represented an important milestone in IC packaging industry as it eliminated the disadvantage of the dual in-line package (DIP) method. Still, delamination is an issue with BGA packaging and merits the attention of researchers.

Our research aims to eliminate the delamination of the low-profile fine pitch ball grid array (LFBGA) and plastic ball grid array (PBGA) package. LFBGA is a thin BGA meeting Joint Electron Device Engineering Council (JEDEC) specifications, with a structure similar to that of conventional BGA, but the diameter and centre of the solder ball are smaller and thinner. Thus, the package contains more I/O numbers within the same size, which signifies that the assembly density of the package is enhanced. Bismaleimide triazine resin or glass layers were used by researchers as a substrate for PBGA packages, due to their similar thermal expansion coefficients [1]. Therefore, when the temperature changes, the IC packaging was less likely to be delaminated. The PBGA was advantageous due to its low-cost. In IC packaging, delamination is an important issue as it occurs due to thermal property mismatches between the various configuration materials [2].

In the development process, contact angle measurements are required to calculate the surface energy of the materials. Materials with high surface energy exhibit hydrophilic characteristics, i.e., the contact angle is small, thus, the materials tend to be highly adhesive. Water is known to spread out almost completely on the surface of noble metals such as gold, silver and copper [3]. Therefore, pure Cu without any oxide layer on the surface is hydrophilic. In the existing studies, CuO film was deposited on a Cu substrate by the successive ionic layer adsorption and reaction (SILAR) method. The CuO thin film showed hydrophobic behavior, with a contact angle of 164° [4]. In addition, the CuO surface may gradually transit to Cu_2_O at room temperature, transforming into a less hydrophobic substance with a contact angle of about 110° [5].

Gas plasma treatment is known to enhance the adhesive properties of materials. In a previous study [6], Cu treated by a plasma cleaning process was used to increase the adhesion strength, where Ar and Ar + H_2_ gases were used as working gases. In another study [7], the effect of low-pressure H_2_ and O_2_ gas plasma cleaning of Au and PtIr was investigated by X-ray photoelectron spectroscopy (XPS). Moreover, Ar and Ar + O_2_ gases were used for plasma cleaning of Au surfaces [8].

In this study, we prepared LFBGA packaging and PBGA packaging materials, performed characterization and determination of physical and chemical properties and analyzed the delamination process. Further, we tried to perform a design optimization which can reduce the delamination phenomena.

**Figure 1:**
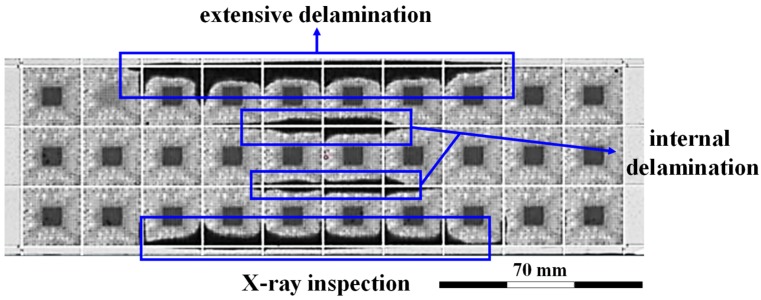
Illustration of delamination in LFBGA packaging process.

To perform the experiments, we took a LFBGA packaged material and observed the SEM image of delamination which was occurring between the metal and the compound. The dark portions, as shown in Figure 1, show delamination. In the semiconductor industry, Cu, Ni, and Cr are commonly used materials in packaging as they show good electrical and thermal properties. Thus, Cu was selected as the substrate in this study, which has a high reference value in the industry. Delamination is defined by the area under delamination expressed as a percentage. For the case of LFBGA, every packaged sample was divided into a 30-cell area (see Figure 1). When one cell has been delaminated, the delamination area percentage (1/30 = 3.3%) was the percentage of the delamination of this 30-cell area. Three different metals were tested to realize the bonding strength between the metals and the compound. Various characterization experiments were conducted to determine the delamination of LFBGA. XPS was used to verify the surface composition under different types of plasma gases. 

**Figure 2:**
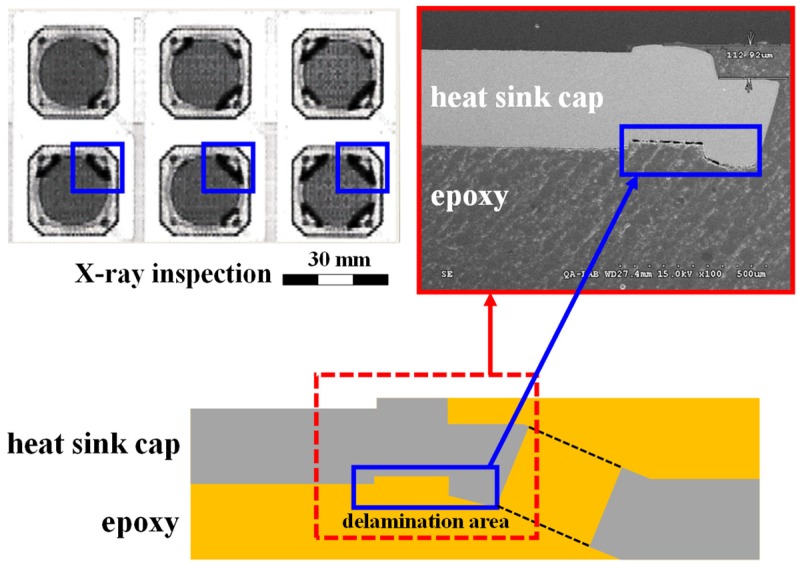
Delamination in PBGA packaging process.

For the case of PBGA packaging, when PBGA was sealed with an epoxy resin, the delamination occurred in the corner of the heat sink cap as shown in Figure 2. Since PBGA was packaged in a high temperature and pressure environment, the shrinkage rate between the heat sink cap and the epoxy resin were different, which deteriorated the adhesion, leading to the delamination. Therefore, ANSYS was used to analyze the stress distribution of PBGA and determine the factors which affected the stress values. Further, full-factorial experiments were designed to optimize the model and decrease the stress values to prevent the occurrence of delamination. 

## 2. Experimental Methods

### 2.1. LFBGA Experiment

The surface morphology and roughness of LFBGA were examined by a SEM and a profilometer, and, the relationship between the delamination percentage and roughness was verified. The delamination in the samples was analyzed and the results were shown in delamination area percentage. The delamination area percentage was classified into 0% to 10% and larger than 10%. A delamination area smaller than 10% is generally acceptable for the clients. 

**Figure 3:**
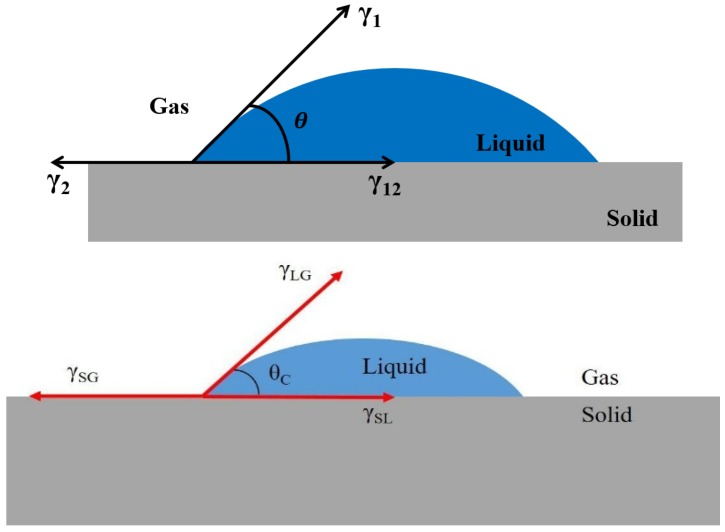
Illustration of contact angle.

The plasma gas of Ar + O_2_ was used to evaluate the effect of the plasma treatment on surface energy, where the gas mixture ratio and operating power were 4:1 and 800 W, respectively. We measured the contact angle after the plasma treatment, based on which, the surface energy and the adhesion can be obtained. The contact angle was used as the method to calculate surface energy. The angle between the solid phase and the liquid phase is defined as the contact angle (θ), as shown in Figure 3. Young’s equation shows that the relation of the adhesion between liquid (γ_1_), solid phase (γ_2_) and the interface of solid and liquid phase (γ_12_) was expressed as Equation (1) [9]. Adhesive-free energy was obtained by Dupre’ equation, as shown in Equation (2) [10]. For the surface energy (surface tension) value was derived by equations (1) and 2, as shown in Equation (3), which is known as Young-Dupre’s equation. The surface energy is correlated with contact angle method. Therefore, this study is focused on the contact angle method to describe the surface energy. Besides, the variation of surface composition before and after plasma treatment, was compared using the binding energy of XPS results, based on the previous references [11,12,13,14,15,16,17,18]. 

**Figure 4:**
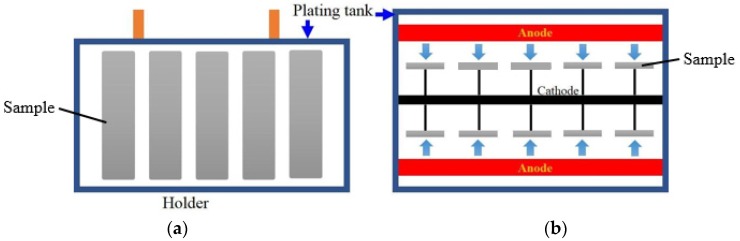

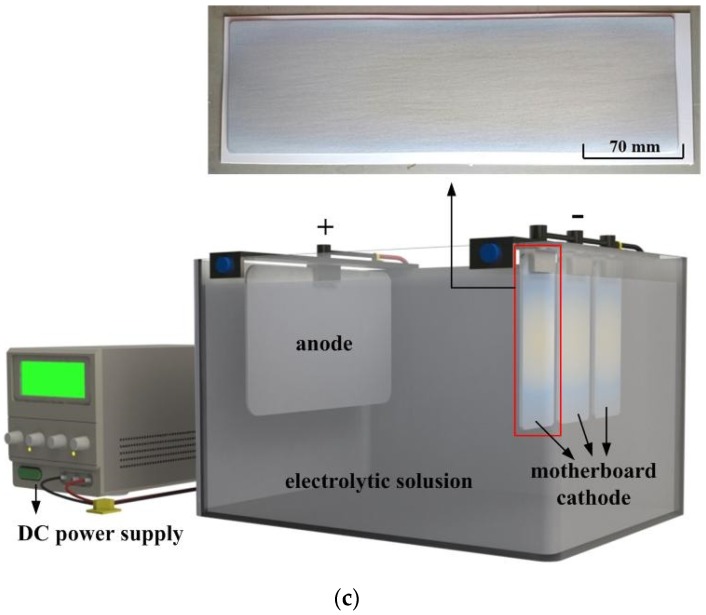
Schematic illustration of electroplated samples (**a**) side view (**b**) top view (**c**) 3D electroplating tank.

Three different metals with varying roughness was electroplated on the Cu substrate, which was functioning as packaging cover. Electroplating process is schematically illustrated in Figure 4. In this study, Cu, Ni and Cr, with two different roughness Ra 3.2 and 3.6, were tested and then the metals were bonded with epoxy to observe the delamination area percentage. The experimental bonding was analyzed to observe the delamination area percentages. The effect of metal type and roughness on delamination were also characterized.

(1) $$\gamma_{2}=\gamma_{12}+\gamma_{1}\cos\theta$$

(2) $$W_{12}=\gamma_{1}+\gamma_{2}-\gamma_{12}$$

(3) $$W_{12}=\gamma_{1}+\gamma_{1}\cos\theta =\gamma_{1}(1+\cos\theta )$$

### 2.2. PBGA Modeling

**Figure 5:**
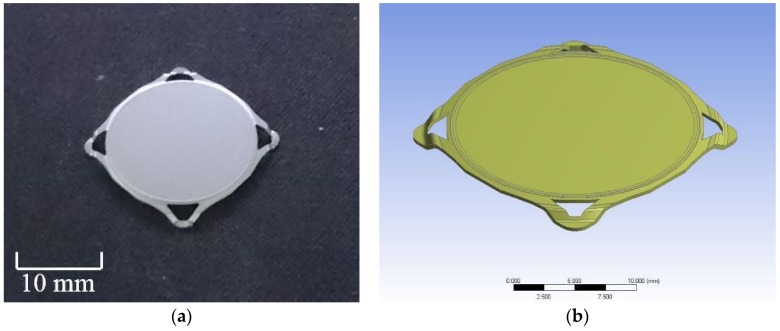
PBGA heat sink cap (**a**) real sample (**b**) model created using ANSYS.

**Figure 6:**
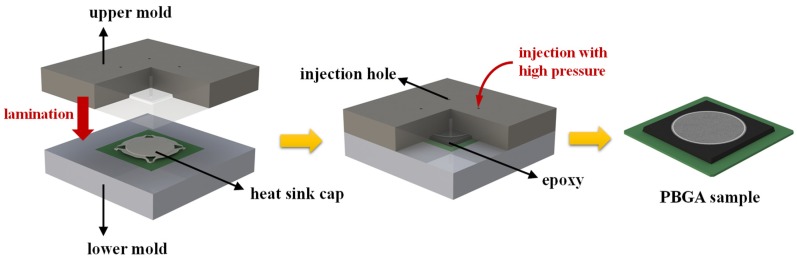
Injection molding illustration of the PBGA packaging process.

The experimental process of PBGA was predicted by using finite element analysis to simulate the effect of the stress distribution on delamination. The geometric parameters of PBGA were provided by Advanced Semiconductor Engineering Inc. (ASE, Kaohsiung, Taiwan). According to the sample information, the heat sink cap is shown in Figure 5a and the model established in ANSYS is shown in Figure 5b. ASE provided the design of the heat sink cap in PBGA package model. During the packaging process, the upper mold covered over the lower mold so that these two molds formed a confined space. The entire confined space was then filled up with epoxy using a high pressure injection tube. The entire model is shown schematically in Figure 6.

### 2.3. PBGA Materials Parameters and Boundary Conditions

**Figure 7:**
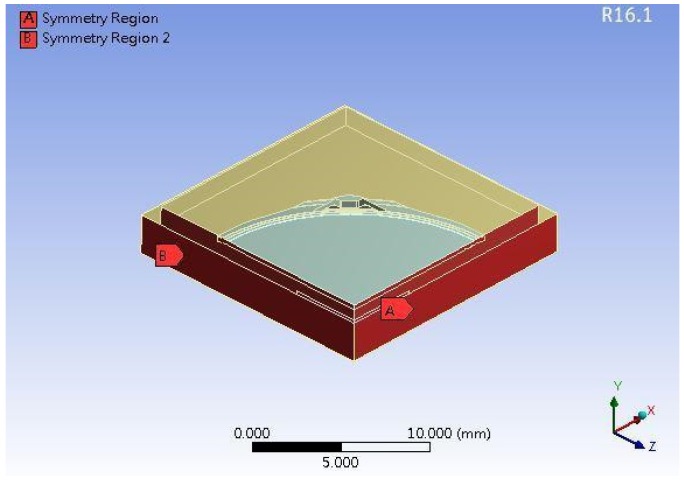
A simplified quarter model for molding simulation.

**Figure 8:**
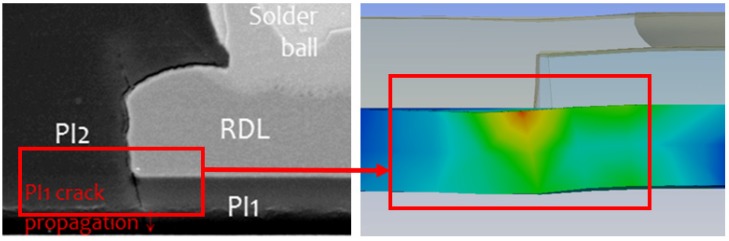
Cross-section morphology of experimental and simulation comparison.

The heat sink cap was made of Cu and the material parameters are listed in Table 1. The *T*_g_ temperature of the epoxy was ~127 °C. There were two thermal expansion coefficients, CTE1 and CTE2 for ANSYS. The boundary condition was set as the upper mold contacting with a circle of ring on the heat sink cap, and lower mold contacting four corners of the heat sink cap. The upper mold and the lower mold were tightly closed, then epoxy was injected into the mold, where the lower mold served as a fixed support. In addition, the simulation molding model was simplified into quarter to reduce the simulation time due to the symmetry, as shown in Figure 7. When the simulation is completed, the program can output the whole model results. This study is focused to develop a simulation model to fast predict the trend of the bonding stress (or delamination stress), beyond which delamination could happen. The delamination stress was correlated with the experimental sample. The cross-section morphology of the experimental sample was compared with the developed simulation model, as shown in Figure 8. The stress at the tip as shown on right hand side in Figure 8 was compared with the experimental sample on left hand side in Figure 8. There was an obvious delamination that happened on the corner.

**Figure 9:**
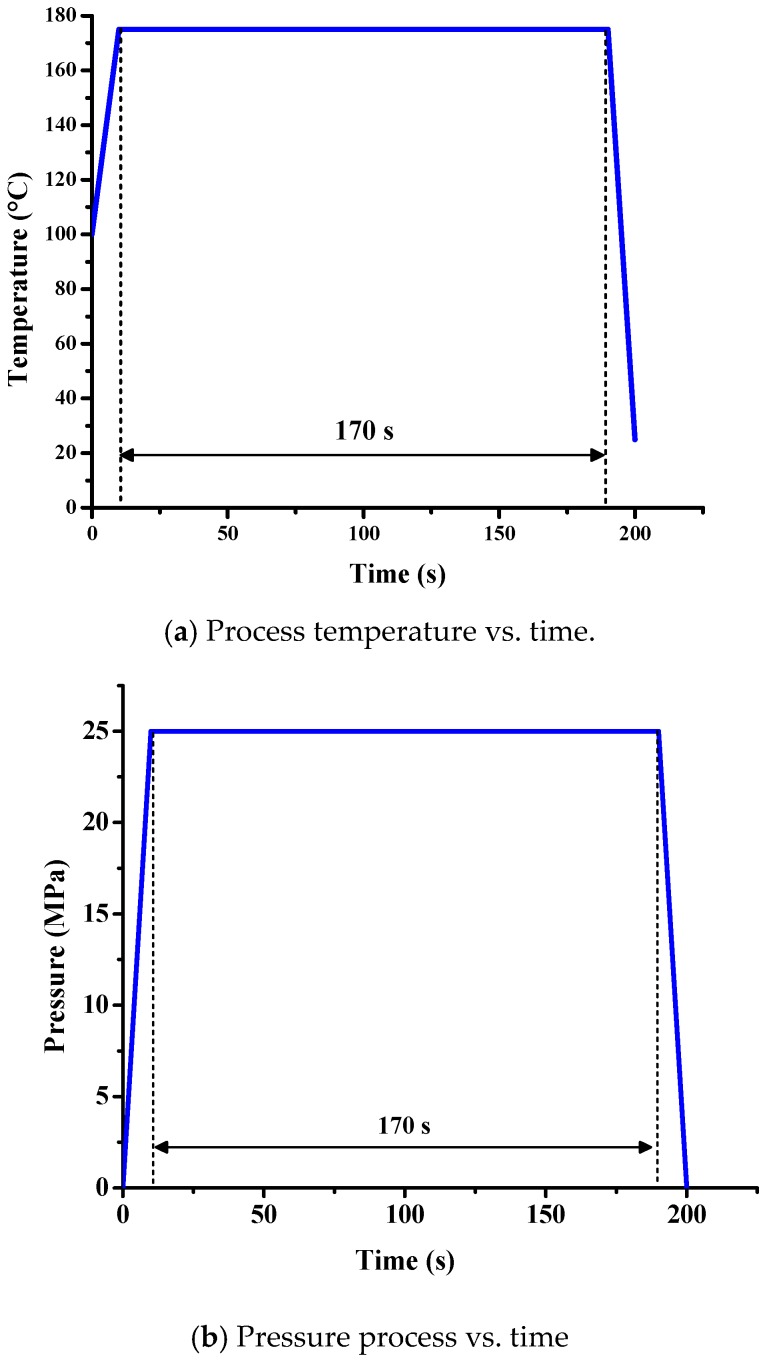
Parameters of (**a**) process temperature and (**b**) pressure for simulation.

The simulation parameter settings of the PBGA packaging are shown in Figure 9. First, the model was preheated at 100 °C, and then the temperature was increased from 100 °C to 175 °C in 10 s. The entire model was maintained at 175 °C for 180 s, and the temperature dropped from 175 °C to room temperature (25 °C) in the last 10 s. For the mold pressure, at first, the upper and lower molds were not contacted. The upper mold was slowly moved downward onto the lower mold with 25 MPa pressure to allow the two molds to be sealed tightly. After 180 s, the upper mold was elevated and the whole packaging process was completed.

### 2.4. PBGA Factors Design

**Figure 10:**
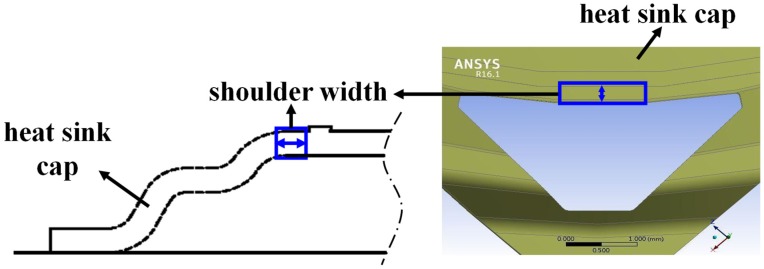
Illustration of the shoulder width.

**Figure 11:**
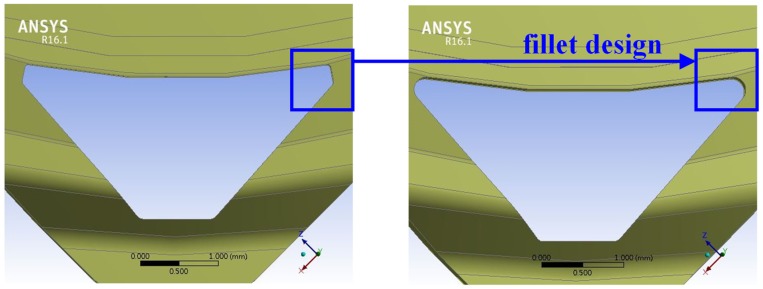
Illustration of the chamfer design.

The main parameters of molding simulation were shoulder width and chamfer radius. The shoulder width is the length next to ring, as schematically shown in Figure 10. The design of the heat sink cap corner with and without chamfer design was considered for the simulation as shown in Figure 11. The original shoulder width was 200 μm, which was set as the lower limit of the parameter, while the upper limit was set as 800 μm. In addition, the lower limit of chamfer radius was 0.15 mm and upper limit of chamfer radius was 0.45 mm, as shown in Table 2. Full-factorial simulations were used to simulate the stress value, as listed in Table 3.

## 3. Results and Discussion

### 3.1. LFBGA Surface Analysis

**Figure 12:**
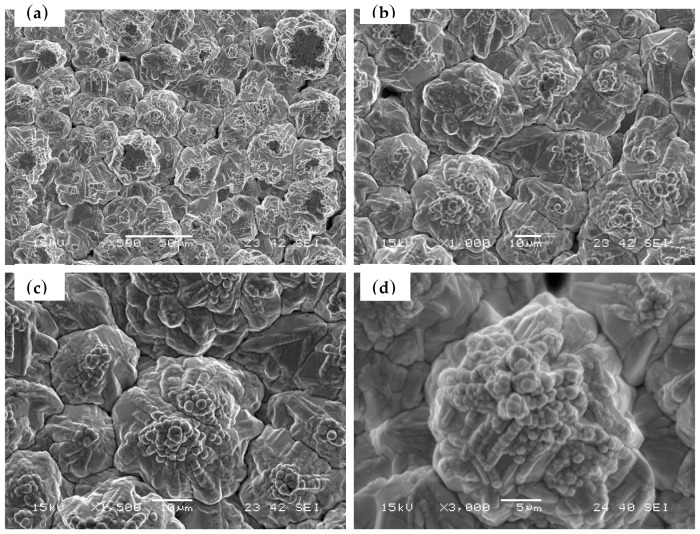
SEM images of the Cu sample (**a**) ×500 (**b**) ×1000 (**c**) ×1500 (**d**) ×3000.

**Figure 13:**
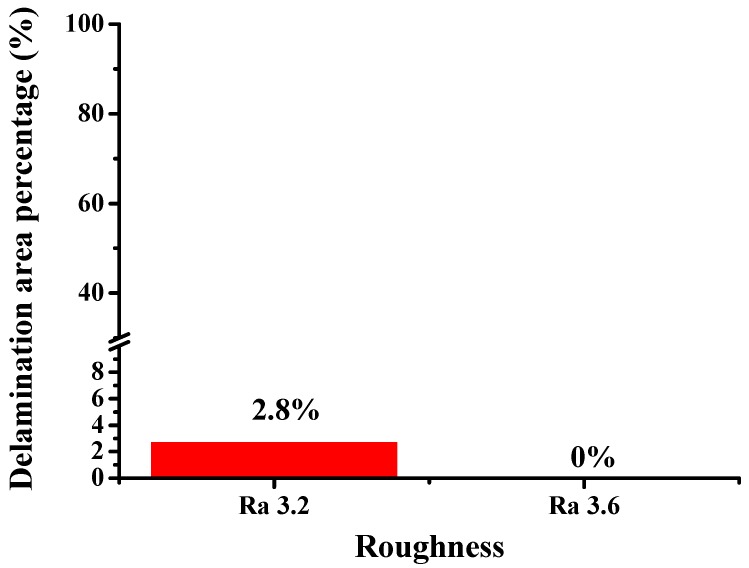
Roughness experiment.

The surface morphology of the LFBGA sample as seen by SEM is shown in Figure 12. A significant roughness of the Cu was observed, which increased the physical adhesion between the metal and epoxy. The relation between roughness of LFBGA and the delamination percentage is shown in Figure 13. The delamination area percentage of roughness Ra 3.2 μm, that is smaller than 10%, accounts for 2.8% of the overall sample; whereas the delamination area percentage of roughness Ra 3.6 μm in the overall sample was zero. In addition, since a delamination area percentage larger than 10% is considered product failure, thus, we focused on the products with the delamination area percentage less than 10%. 

When the roughness increased from Ra 3.2 μm to Ra 3.6 μm, the delamination percentage decreased from 2.8% to 0.0%. This decrease was due to the increasing roughness and subsequently enhanced physical adhesion and the bonding force.

### 3.2. LFBGA Plasma and XPS Analysis

**Figure 14:**
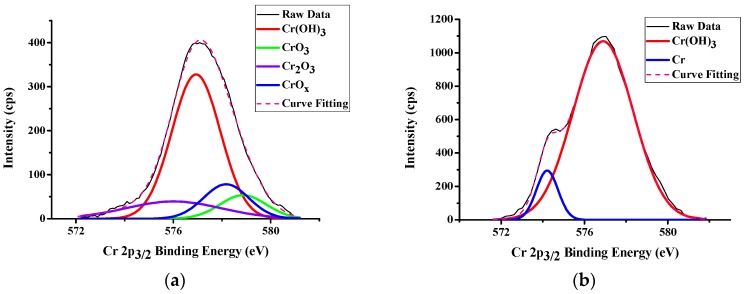
XPS results of Cr (**a**) Before Ar + O_2_ plasma treatment (**b**) After Ar + O_2_ plasma treatment.

**Figure 15:**
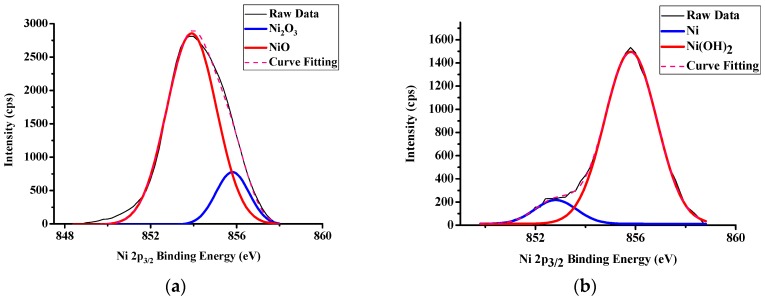
XPS results of Ni (**a**) Before Ar + O_2_ plasma treatment (**b**) After Ar + O_2_ plasma treatment.

**Figure 16:**
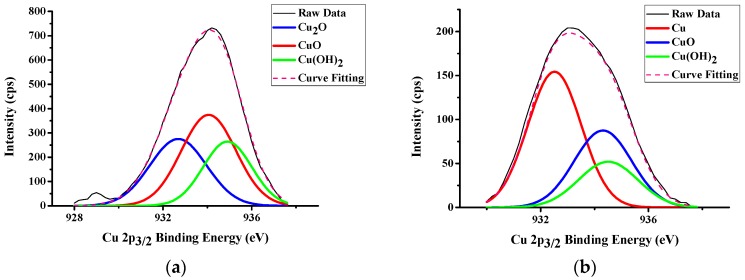
XPS results of Cu (**a**) Before Ar + O_2_ plasma treatment (**b**) After Ar + O_2_ plasma treatment.

Three different metals were treated with Ar + O_2_ plasma to clean the surface and the results of XPS are shown in Figure 14, Figure 15 and Figure 16, respectively. The Cr metal surface before plasma treatment is shown in Figure 14a, where oxide layers exist on the surface. However, after the plasma treatment, the oxide layers were removed by plasma particles, as shown in Figure 14b, because the plasma particle bombarded the surface oxide layer. In addition, the differences between before and after plasma treatment of Ni are shown in Figure 15. The XPS results were similar to those of Cr, since the oxide layers were also removed by plasma particle after the plasma treatment. In Cu metal, there were oxide on the surface, and after the plasma treatment, the amount of oxide was decreased. Still, a small amount of oxide layers remained on the Cu surface as shown in Figure 16. The reason is that the Ar gas can remove the oxide layers, but Cu might react with O_2_ gas and might produce CuO and Cu_2_O compounds. 

### 3.3. LFBGA Contact Angle and Different Metals Analysis

**Figure 17:**
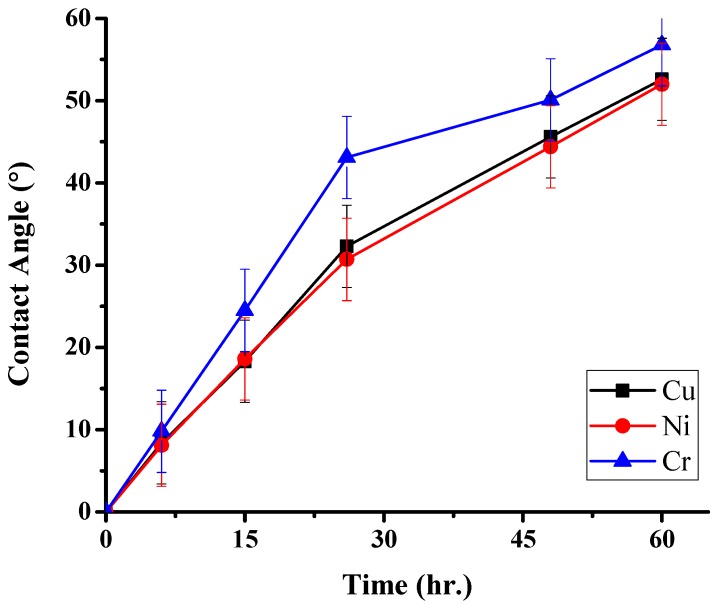
Contact angle vs. time after plasma treatment of Cr, Ni and Cu, respectively.

Based on a previous study [4], it is seen that the oxide layer is hydrophobic and the surface energy is also low, which are disadvantageous in IC packaging. In this study, the results of contact angle of three metals were measured, and shown in Figure 17. After Ar + O_2_ plasma treatment, the metal surface became hydrophilic, and the water was completely stuck on the surface because the oxide layer was removed. After 60 h of Ar + O_2_ plasma treatment, the contact angle of Cu was increased to about 52.6° since oxide layers were growing on the surface, which may make the surface gradually hydrophobic, and thus, cause an increase in the contact angle. It means that after being treated for a period of time, the treated sample could lose hydrophilicity. The contact angle of Ni was increased to 52° and Cr was increased to 56.8°, however, the error bars of three metals were overlapping. Therefore, the differences of contact angle in three metals were not statistically significant.

**Figure 18:**
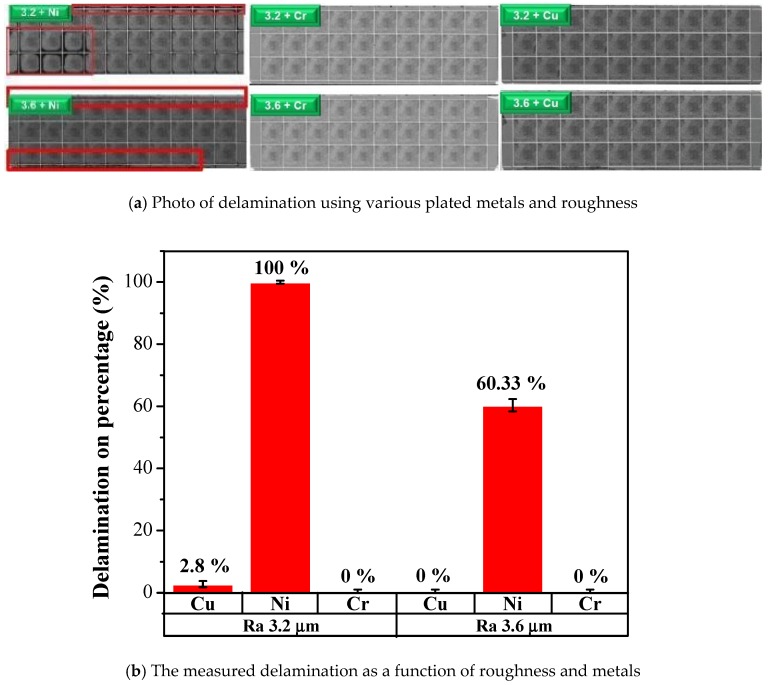
The effect of different metals and roughnesses on delamination.

The results of the various metals with different roughness of Ra 3.2 and Ra 3.6 is shown in Figure 18. The roughness was measured by a SJ-400 profilometer (Mitutoyo, Kawasaki, Japan). 

When Cu metal was bonded with epoxy, the delamination percentage was ~0–2.8 % for Ra 3.6 and 3.2, respectively. However, the delamination was 60.33% when Ni metal plate had Ra 3.6, whereas the delamination increased to 100% when Ra was down to 3.2. The Cr metal exhibited similar results to the Cu samples. Almost no delamination took place. Through these results, epoxy showed good bonding force with Cu and Cr. However, Ni did not have adequate bonding force with epoxy, which led to the delamination. In other words, after electroplating, the surface of the Ni layer was supposed to prevent the occurrence of copper oxidation and increase the adhesion with polymer. Since the Ni layer after electroplating could be less adhesive than Cr layer, O_2_ could get into the Cu substrate from the Ni layer and form CuO and Cu_2_O compounds simultaneously, causing an uneven stress distribution beneath the Ni layer. Delamination could occur between polymers and Ni after packaging. Epoxy adhesion with Ni was weaker than with those of Cr or Cu. Therefore, in order to decrease the delamination, the substrate should not be coated with Ni metal.

### 3.4. PBGA Simulation

**Figure 19:**
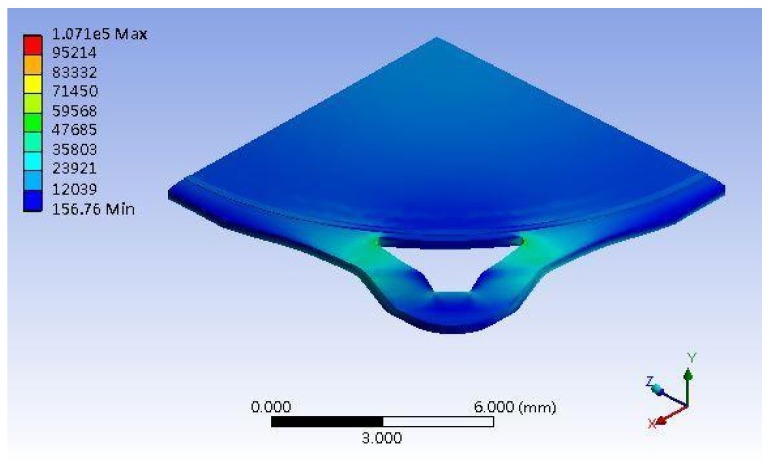
ANSYS simulation result.

**Figure 20:**
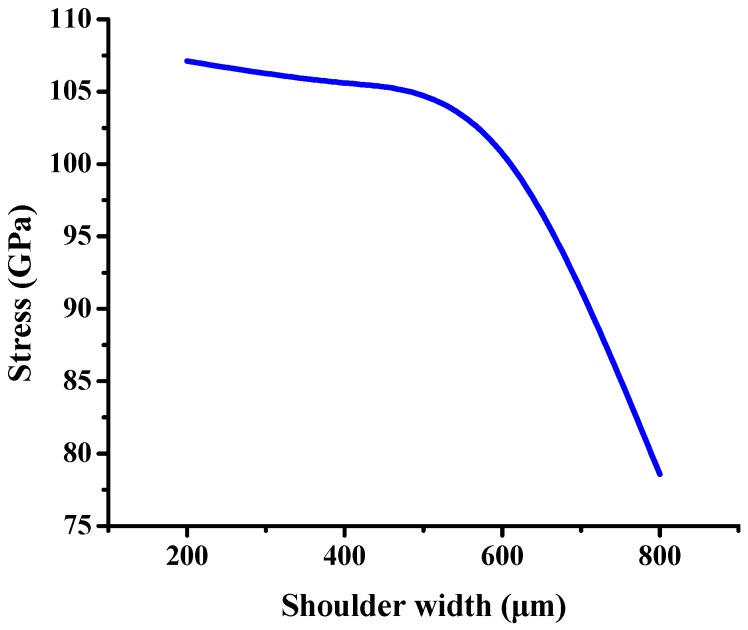
Stress vs. shoulder width.

The molding simulation result of PBGA heat sink cap is shown in Figure 19. The stress is concentrated at the corner of the heat sink cap where delamination occurred with a stress value of ~107.1 GPa. One factor of the shoulder width was changed to simulate the stress value. The results of different shoulder widths are shown in Figure 20. When the shoulder width increased up to 800 μm, the heat sink cap generated the lowest stress value. 

As shown in Figure 20, the stress value was inversely proportional to the shoulder width. The location of shoulder is next to the ring, where the upper mold applied pressure. When the shoulder width increased, the stress concentration was released and the delamination issue was eliminated. The maximum of shoulder width is no more than 800 μm in consideration of product size. If the shoulder width is larger than 800 μm, the heat sink cap cannot maintain the original size. Therefore, the maximum shoulder width was set as 800 μm.

**Figure 21:**
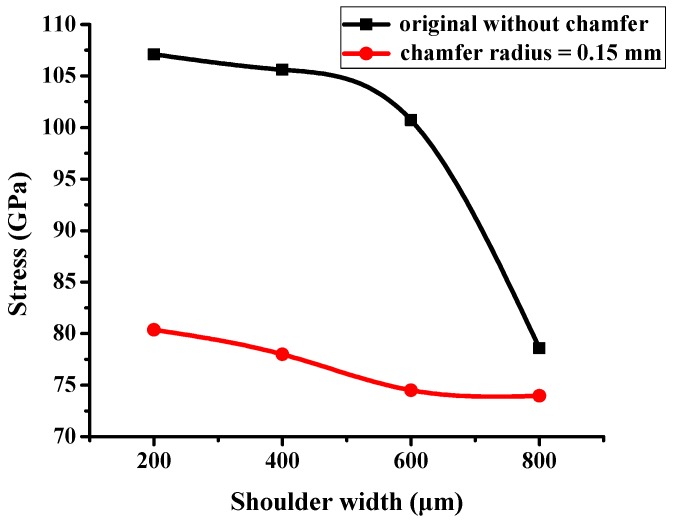
Comparison between the original design and the chamfer design.

The original design with a sharp geometry of the heat sink cap corner was not an ideal and optimal case, which led to a significant stress concentration. Therefore, the new design has changed the sharp geometry to chamfer with various radii. The comparison result between original and chamfer design with radius 0.15 mm is shown in Figure 21. The stress value of chamfer design was significantly smaller than the original design. Adding chamfer design exhibited a smaller stress which might be due to the chamfer releasing the stress concentration at the corner. In addition, the maximum radius of chamfer was 0.45 mm as when the radius is larger than 0.45 mm, the geometry of the corner for epoxy to flow in could be changed. Therefore, there is a limit for radius of chamfer, which is 0.45 mm.

**Figure 22:**
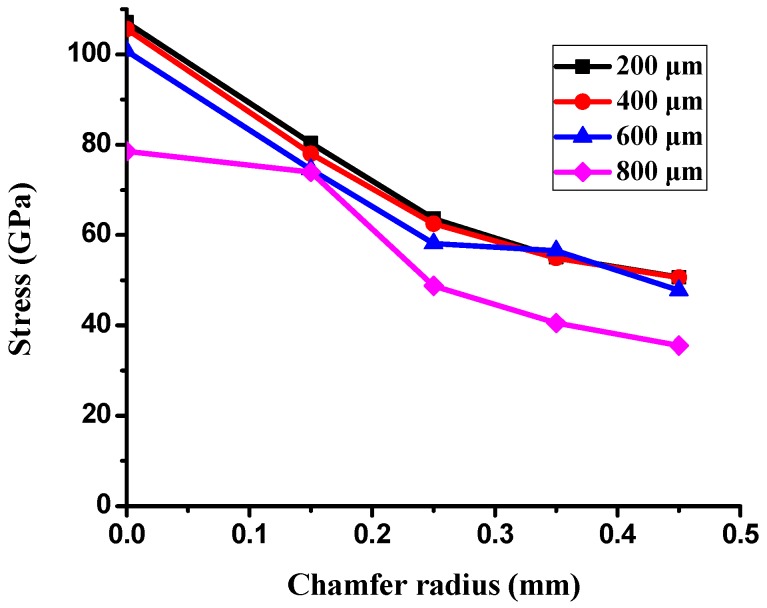
Stress vs. chamfer radius and shoulder width.

In addition, the chamfer design of four different radii combined with the various shoulder widths to simulate the stress value. The result shows that when the radius and the shoulder width increased, the stress values were decreased (see Figure 22). Therefore, the delamination was eliminated after the shoulder width increased and the chamfer radius was added into the new design. When the radius of chamfer was 0.45 mm, the stress values decreased significantly. Moreover, when the shoulder width was 800 μm, the stress displayed a minimum value of 35.58 GPa.

## 4. Conclusions

From the aspects of physical and chemical properties, the delamination problems of LFBGA and PBGA were discussed in this study. The roughness has a significant influence on delamination, and it is inversely proportional to delamination. The delamination was improved when Ra was changed from 3.2 to 3.6 μm. Based on the XPS results, it can be observed that Ar or N_2_ gas plasma could remove most of the oxide layer and increase the surface energy. On the other hand, it could also induce the oxide layer to grow. The original state of three kinds of metals had oxide layers on the surface, but after Ar + O_2_ plasma treatment, most of the oxide layers were removed and the contact angles were decreased, which is suitable for IC packaging. The reason why the delamination between Ni and epoxy occurred could be the weak chemical bond formed between Ni and the epoxy compound materials. The Ni layer after electroplating could be less adhesive than the Cr layer, and O_2_ could get into the Cu substrate from the Ni layer and form CuO and Cu_2_O compounds simultaneously, causing the uneven stress distribution beneath the Ni layer. The composition of the epoxy was not known well at this stage due to the secrecy of the supplier, which will be studied further in the near future. However, Cu and Cr metal bonded well with the epoxy. As the shoulder of the PBGA heat sink cap was increased from 200 to 800 μm, the stress was decreased from 107 to 78.57 GPa. Under the same conditions, the geometry design was further improved and adding the chamfer effectively improved the stress value, dropping it from 107.1 to 80.37 GPa. When the shoulder width of the heat sink cap was changed to 800 μm and chamfer radius to 0.45 mm, the stress value was decreased from the original stress value of 107 to 35.58 GPa. The reduction in stress value indicated that the delamination between metal and epoxy has been improved significantly.

## Figures and Tables

**Table 1:** Material parameters.

Material Parameter	Cu	Epoxy	Units
**Density**	8940	1.75	kg/m^3^
**Young’s modulus**	117	25.48	GPa
**Poisson’s ratio**	0.3	0.3	--
**Heat transfer coefficient**	391	--	W/m·K
**Specific heat**	385	--	J/kg·K
**CTE**	16.5	9 (CTE1), 34 (CTE2) (T_g_: 127 °C)	ppm/K

**Table 2:** Lower and upper limits of parameters.

Mechanical Parameter	Lower Limit	Upper Limit	Units
**Shoulder width**	200	800	μm
**Chamfer radius**	0.15	0.45	mm

**Table 3:** Full-factorial simulation.

Group	Shoulder Width (μm)	Chamfer Radius (mm)
1	200	Original without chamfer
2	200	0.15
3	200	0.25
4	200	0.35
5	200	0.45
6	400	Original without chamfer
7	400	0.15
8	400	0.25
9	400	0.35
10	400	0.45
11	600	Original without chamfer
12	600	0.15
13	600	0.25
14	600	0.35
15	600	0.45
16	800	Original without chamfer
17	800	0.15
18	800	0.25
19	800	0.35
20	800	0.45
